# Computer-assisted beat-pattern analysis and the flagellar waveforms of bovine spermatozoa

**DOI:** 10.1098/rsos.200769

**Published:** 2020-06-17

**Authors:** Benjamin J. Walker, Shiva Phuyal, Kenta Ishimoto, Chih-Kuan Tung, Eamonn A. Gaffney

**Affiliations:** 1 Wolfson Centre for Mathematical Biology, Mathematical Institute, University of Oxford, Oxford OX2 6GG, UK; 2 Department of Physics, North Carolina A&T State University, Greensboro, NC 27411, USA; 3 Research Institute for Mathematical Sciences, Kyoto University, Kyoto, 606-8502, Japan

**Keywords:** flagellar analysis, spermatozoa, computer-assisted sperm analysis

## Abstract

Obstructed by hurdles in information extraction, handling and processing, computer-assisted sperm analysis systems have typically not considered in detail the complex flagellar waveforms of spermatozoa, despite their defining role in cell motility. Recent developments in imaging techniques and data processing have produced significantly improved methods of waveform digitization. Here, we use these improvements to demonstrate that near-complete flagellar capture is realizable on the scale of hundreds of cells, and, further, that meaningful statistical comparisons of flagellar waveforms may be readily performed with widely available tools. Representing the advent of high-fidelity computer-assisted beat-pattern analysis, we show how such a statistical approach can distinguish between samples using complex flagellar beating patterns rather than crude summary statistics. Dimensionality-reduction techniques applied to entire samples also reveal qualitatively distinct components of the beat, and a novel data-driven methodology for the generation of representative synthetic waveform data is proposed.

## 1. Introduction

In the context of analysing spermatozoa, information handling and processing has, in general, substantially lagged behind advances in microscopy. For instance, phase contrast microscopy was developed and commercialized in the 1930s and 1940s [1] and enables a straightforward visualization of the spermatozoan flagellum, but the prospect of capturing and using the wealth of flagellar information beyond hand tracings [2] was not feasible until the 1980s, with software packages such as ‘BohBoh’ introducing a degree of automation [3]. However, the extensive user intervention still required for such packages and early thresholding-based techniques, for example by Smith *et al.* [4], who reported on the beats of 36 swimmers, has to date largely restricted population-level computer-assisted sperm analysis (CASA) to head-based investigations [5–8] and assessments of flagellar morphology [9], which largely neglect the details of flagellar waveforms, although contributing significantly to the understanding of spermatozoa at the time. Despite its only partial acceptance in clinical diagnostics [10], CASA has also found extensive application in reproductive toxicology, quality assurance for semen marketing in livestock breeding, improvements in sperm technologies such as cryopreservation, and studies of basic sperm function [11–14].

Furthermore, digital videomicroscopy that captures flagellar movement potentially contains a wealth of additional information, motivating the need for more user-friendly, less user-intensive techniques to capture and analyse the moving flagellum. A recent tool has been developed for this purpose with phase contrast microscopy by Gallagher *et al.* [15], though it is limited due to its current inability to capture much of the distal region of the flagellum, despite the reported importance of the distal flagellum in motility mechanics [16]. Alongside similar studies of the biflagellated alga *Chlamydomonas reinhardtii* [17–19], other recent works have developed techniques to capture the beating flagellum [20], including the distal region, most notably the three-dimensional capture of the flagellar waveform achieved by Daloglu *et al.* [21] with holographic imaging. However, compared to phase contrast microscopy, this is a highly specialist imaging modality requiring custom circuitry together with sophisticated hardware and processing, while the former is already ubiquitous in theriogenology and andrology facilities [6,9], and also in the study of a wealth of eukaryotic monoflagellates [22].

Proposing a different basis for flagellar capture, the automated methodology of Walker *et al.* [23] uses the approximately consistent width of the flagellum to distinguish it from the head of the swimmer, not relying on explicit image contrast between cell components and thus potentially applicable to typical videomicroscopy even with low signal-to-noise ratios, with far less user-processing than required with many systems. Thus, as an initial aim of this work, we seek to make available a large dataset of near-complete flagellar waveforms for hundreds of motile bovine sperm cells via the existing methodology of Walker *et al.* [23], in doing so highlighting that the fundamentals of a new generation of *computer-assisted beat-pattern analysis* (CABA) for essentially the whole and motile flagellum are realizable for eukaryotic monoflagellates on current, widely available hardware using simple, intuitive image processing.

While the ability to readily document the flagellar beat in high fidelity using commonly available imaging modalities has significantly broadened the potential scope of CASA, arguably of greater importance is the prospect of detailed quantitative analysis incorporating the entirety of the motile flagellar waveform. Such statistical analyses, with the power afforded by non-small sample sizes, would provide the capabilities required for a new generation of swimmer evaluation studies, based on quantitative assessment of not only coarse summary statistics, as is the current standard [6,8], but also the complex time-evolving shape of the flagellar beat. However, even given detailed waveform data, there is no clear or established methodology for performing statistical comparisons and hypothesis testing on flagellum data.

In numerous previous works [24–27], the waveforms of individual beating flagella have been simplified via dimensionality-reduction techniques, a pertinent example being the principal component analysis (PCA) employed by Ma *et al.* [26] though not in a statistical context, with seven swimmers analysed. This standard technique aims to decompose a dataset into a small number of representative modes, capturing the variance of the underlying observations. Here, building upon captured waveform data, we will adopt such dimensionality-reduction techniques, though notably in novel application to entire samples, to generate distributions that describe the flagellar waveform and its variation across a sperm population. The primary and fundamental objective of this work will then be to present a methodology for quantitative comparative waveform analysis, using these population-level distributions to enable flagellar-based sample comparisons via the subsequent application of established techniques for statistical hypothesis testing in this novel context. In particular, these techniques will be illustrated by considering the beating patterns of post-thaw spermatozoa that present with distal cytoplasmic blebbing, assessing whether or not their waveforms may be distinguished from those unaccompanied by signs of damage. In turn, this showcases how computer-assisted beat-pattern analysis may be used to quantitatively compare spermatozoan flagella across populations.

Finally, a secondary objective of this study will be to use the captured waveform data in the generation of evidence-based synthetic flagellar waveforms by a simple sampling method. In doing so, we will provide a methodology for the construction of arbitrary numbers of evidence-based synthetic flagellar waveforms for use in biophysical population modelling, with current modelling frameworks accommodating hundreds [28] and even up to 1000 [29] virtual spermatozoa. In turn, this will enable future studies to assess the physical consequences of evidence-based population variability in explorations of flagellated swimmer active matter without being constrained to make only direct use of captured waveform data, and instead able to use innumerable waveforms representative of a population.

In summary, we will generate and present a large dataset comprising the fine details of swimmer waveforms from phase contrast videomicroscopy, using widely available non-specialized hardware and simple, effective image processing. Equipped with rich kinematic data, we attempt to decompose the flagellar beat into its most prominent components, highlighting a data handling methodology that can realize the potential for high-fidelity flagellum datasets such as that presented here to facilitate biological inquiry at population level into spermatozoan flagellar dynamics. Further, and most significantly, we will explicitly demonstrate by example that detailed qualitative comparison of samples based on highly resolved waveform information is a realizable direction for CASA, potentially underpinning a new generation of automated flagellum analysis. Finally, we will then leverage the presented dataset to produce synthetic waveform data, proposing a novel method of beat pattern generation that captures key features of the spermatozoan beat, envisaging its use in future biophysical modelling applications.

## 2. Results

### 2.1. Large-scale quantification of flagellar beating

**Figure 1.**
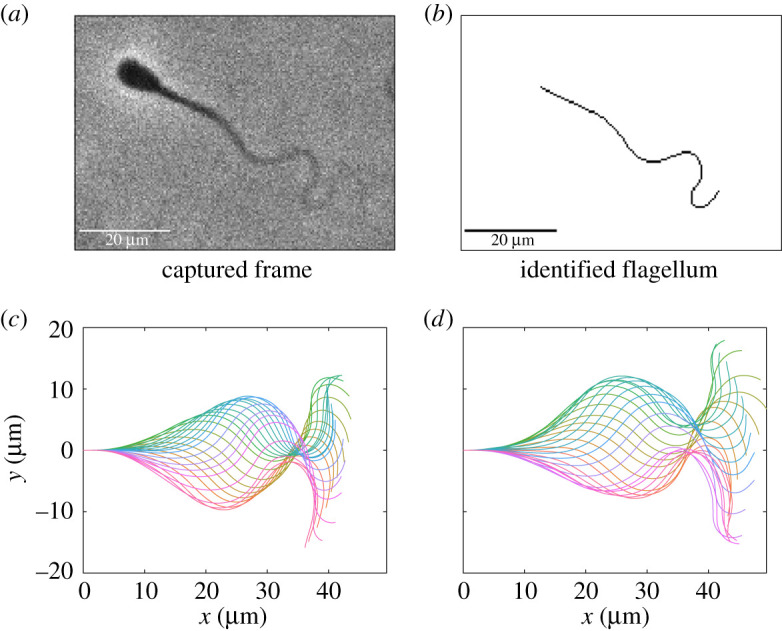
A sample captured frame from a fresh spermatozoon, the identified flagellum and complete waveform, accompanied by a synthetic waveform constructed from the presented dataset using the method of generation proposed in §2.4 with *N*_*s*_ = 10. (*a*,*b*) Raw image data alongside the flagellum as identified using the automated methodology of [23], with the vast majority of the flagellum being captured. (*c*) A captured waveform over a single beating period, with a sample synthetic beat shown in panel (*d*) generated following equation (2.2), drawing flagellar arclength and beating period from their respective empirical distributions. In panels (*c*,*d*), plotted in different colours are the shapes of the flagella at select timepoints within one period, with the base of each flagellum situated at the origin of their respective Cartesian coordinate systems. We remark that these waveforms are qualitatively similar, with the proposed sampling method replicating the mean and variance of the empirical waveform distribution by construction. The waveforms in panels (*c*,*d*) have been rescaled by the original and sampled flagellum lengths in order to enable comparison to imaging data.

We imaged the beat patterns of swimming bovine spermatozoa in 1% methylcellulose dissolved in Tyrode albumin lactate pyruvate (TALP) medium, and digitally captured approximately 90% of the planar-beating flagellum. This dataset is freely available (as detailed in the data access statement), and presents spatially and temporally smoothed parametrizations of the flagellar waveforms for 216 swimmers, given over a single period, aligned in phase, and normalized as described in the Methods and material. We present a sample frame, identified flagellum and a captured beat pattern in figure 1. Our dataset represents the digitization of flagellar waveforms not previously captured on this scale via the common imaging modality of phase contrast, with previous distal capture limited at approximately 70% [15]. We present this dataset of automatically captured and smoothed flagellar beats in both the Cartesian and tangent angle forms (see Methods and material), accompanied by the computed beating period and truncated flagellum length. We also provide raw tracking data generated by the software TrackMate [30], which may be used in isolation to compute the wide range of existing CASA measures [8]. As detailed fully in the electronic supplementary material, swimmers are stratified into two samples based on source and handling, which are referred to as samples A and B throughout.

### 2.2. Variation across spermatozoa

With hundreds of spermatozoa imaged and their flagellar waveforms digitized, we perform several exemplar comparisons of the flagellar beat between the two different samples. This highlights that meaningful comparison between swimmers is not simply limited to coarse summary statistics and head tracking, but may take into account the fine details of the flagellar waveform. Innumerable additional comparisons are possible in future studies given the presented dataset and the CABA methodological framework, with sample sizes providing statistical power and flagellum detail enabling the exploration of novel beat metrics. We note that, due to the spermatozoa in samples A and B originating from different individuals and having been subsequently handled differently, as detailed fully in the electronic supplementary material, we do not seek to draw biological insight from the comparisons performed below, with these intersample comparisons nevertheless serving to exemplify the comparative methodology that is the principal focus of this study.

#### 2.2.1. Quantitative waveform comparison

**Figure 2.**
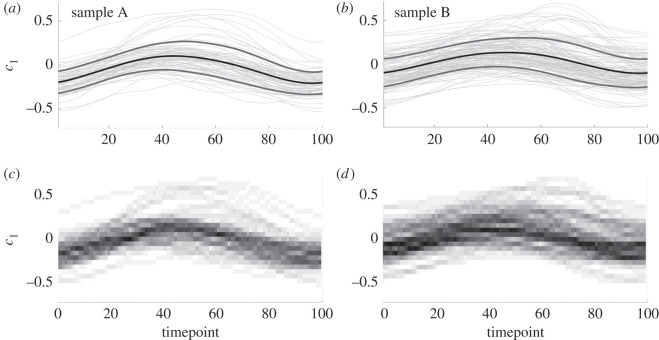
Distributions of the first Cartesian PCA coefficient, *c*_1_, over the course of a single beat, with samples A and B shown in the left and right columns, respectively. (*a*,*b*) The traces of *c*_1_ for each swimmer as light grey curves, accompanied by the sample mean, presented as a black, heavy curve. Shown also is the mean plus or minus a single standard deviation, here drawn as heavy grey curves flanking the mean. (*c*,*d*) The empirical distributions of the first PCA coefficient, constructed by computing a histogram at each timepoint and associating each bin probability with a greyscale value that is uniformly varying on the interval [0, 1]. Darker regions correspond to higher-density regions of the histograms, with the modal values approximately following the mean curves shown in (*a*,*b*), consistent with approximate normality. Visual comparison between samples is difficult, though increased variance appears to be present in sample B compared to sample A between timepoints 40 and 80. In both samples, we recognize an approximate sinusoidal shape of the mean and mode, suggesting that a simple sinusoidal fit may be appropriate to capture the evolution of the first PCA coefficient over time.

Performing PCA *on the entire population of swimmers* in Cartesian form, as detailed in the Methods and material, we can interrogate the resulting coefficients to identify any significant differences in the beating patterns of samples A and B, with the first three PCA modes cumulatively capturing 90% of the population variance. In figure 2, we present the distribution of the coefficient of the first Cartesian PCA mode for each individual normalized waveform, separated by sample and shown over the course of a single beat. Shown in figure 2*a*,*b* are the time series of the first PCA coefficient, *c*_1_, for each swimmer, accompanied by the evolution of the sample means plus and minus the standard deviation (shown as darker, heavier curves). To the resolution shown in the figure, sample B appears to display larger variance during the central 40 timepoints of the beating than sample A, in addition to an increased mean value. This is more apparent in figure 2*c*,*d*, which for each timepoint show the coefficient distribution as a colour-coded histogram, where higher values of the empirical probability distribution are shown as darker regions. The higher variance of sample B can be observed, in addition to the consistently unimodal nature of the distribution of coefficients over swimmers.

**Figure 3.**
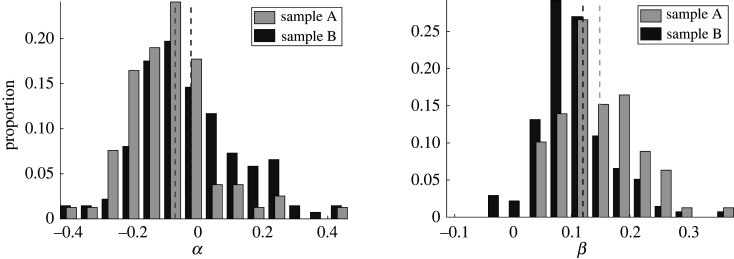
Marginal empirical distributions of fitted parameters (see equation (2.1)), stratified by sample. Having fit the coefficients of a simple sinusoidal model to *c*_1_, as suggested by figure 2, we show the empirical distributions of the fitted parameters as histograms, with those corresponding to sample B shown darkest. Visual differences between samples in median (shown as dashed lines) and variance are evident by eye for each of the fitted parameters, supported by Kolmogorov–Smirnov tests at the 5% significance level. Thus, we can quantitatively distinguish between the flagellar beating of two samples of spermatozoa.

Further to qualitative comments that speculate differences between the two samples, we may also conduct quantitative comparisons. Owing to the unimodality of the distributions and the simple temporal form of the means of the first PCA coefficient, we may compare the fits of a linear model for PCA mode coefficients via the distributions of the linear fitting parameters in order to examine statistical differences between samples. With the form and details of the linear model presented in the Methods and material, in summary we find that simple sinusoidal fits to the dataset reveal a statistically significant difference between the two samples, in concurrence with the difference in means that can be seen between the left and right columns of figure 2, though such a difference is hard to detect, and especially quantify, by eye. In figure 3, we explicitly show empirical histograms for the parameters *α* and *β* in a statistical fit of *c*_1_ to a sinusoidal function

**Figure 4.**
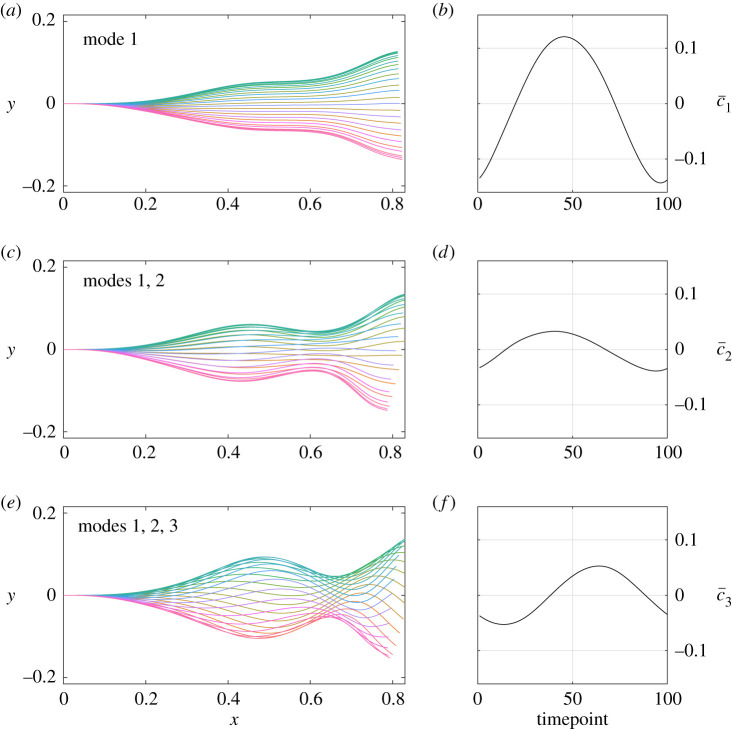
The cumulative contributions of the most prominent Cartesian PCA modes, added to the population mean. (*a*,*c*,*e*) The effects of the three most significant modes, presented as the normalized flagellar waveforms generated by the modes along with the average Cartesian PCA coefficients, shown over a single beating period and with the base located at the origin. These average PCA coefficients are denoted by ${\bar{c}}_{i}$ for *i* = 1, 2, 3, with their evolution shown over time in (*b*,*d*,*f*). We can see that the dominant mode, capturing 75% of the population variance over a beating period, prescribes the amplitude and overall shape of the flagellar beat, with the maximum of ${\bar{c}}_{1}$ greater in magnitude than the other coefficients. The contributions of the higher-order modes are out of phase with the primary mode and of reduced magnitude, though we note that the third mode appears to generate the recognizable sinusoidal shape that is characteristic of many bovine spermatozoan beat patterns. Different colours correspond to different timepoints throughout the flagellar beat, consistent between rows. Without loss of generality, all modes and coefficients have been normalized to enable direct comparisons between PCA coefficients.

2.1 $$c_{1}=\alpha +\beta \cos\left(kt-\phi \right),$$

where wavenumber and phase parameters *k* and *ϕ* are first approximated by nonlinear least-squares fitting of the mean coefficient, ${\bar{c}}_{1}$ (See figure 4, and the section entitled ‘Statistical tests’ within Methods and material for further details). In particular, such tests facilitate detecting and testing for differences between sample A and sample B more readily. Thus, via consideration of the details of the flagellar waveform, subtle but significant differences between samples may be both automatically identified and rigorously quantified, using an approach that may be readily extended to include additional PCA coefficients and alternative methods of dimensionality reduction.

#### 2.2.2. Beating period

**Figure 5.**
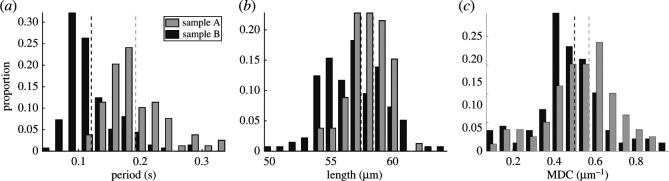
Empirical distributions of beating period, flagellum length and maximum distal curvature (MDC), shown as histograms and separated into samples A and B. (*a*) Distributions of the period of the flagellar beat, from which we can visually identify differences between the samples. Though less pronounced, significant differences between both medians and entire distributions can also be seen for the observed flagellum length and the maximum distal curvature, shown in (*b*) and (*c*), respectively. The medians of each distribution are shown as dashed vertical lines, with distributions corresponding to sample B shown darker, and all differences in distribution reported here are confirmed via Kolomogorov–Smirnov tests at the 5% significance level, with medians similarly assessed by the Wilcoxon rank-sum test.

A fundamental feature of any periodic beating is the extent of its period, which we briefly consider due to its ubiquity. We define the period as the time interval after which the flagellum most closely resembles its initial configuration, matching our intuitive notion of a beating period, as detailed in the Methods and material. In figure 5*a*, we report the period of the flagellar beat for each of the individuals in samples A and B, with the medians shown as dashed lines. The two distributions appear starkly distinct to the eye, supported by a Wilcoxon rank-sum test at the 1% significance level, in addition to a significant Kolmogorov–Smirnov test statistic.

#### 2.2.3. Flagellum length

Now calculable to greater accuracy than previously attainable on large scales, figure 5*b* shows the distribution of flagellum length for each sample. We again see statistically significant differences between the distributions, in both median and overall distribution. In line with noted morphological homogeneity in bovine spermatozoa [31], apparent from the distributions of arclength is the low variance about the mean, true of both samples individually and when combined (not shown). With a coefficient of variation of less than 4%, such tight spread supports our rescaling of all flagella by total length, enabling comparison by principal component analysis.

#### 2.2.4. Extremes of distal bending

With such extensive flagellar detail captured, we consider the previously unavailable metric of maximal distal curvature (MDC), suggested by Neal *et al.* [16] to be correlated with swimming efficiency. We compute the MDC of each of the swimmers throughout their beat, as described in the Methods and material, which are shown as histograms in figure 5*c* stratified by sample. We note statistically significant differences between the two samples, consistent with the PCA analysis above and highlighting how fine details of the flagellar beat can be used to distinguish populations of swimmers in terms of suggested surrogates of functional performance, such as swimming efficiency.

#### 2.2.5. Contributions to the flagellar beat

Population-level principal component analysis allows us to quantify the components of the flagellar beating of bovine spermatozoa, accounting for variation throughout the individual beats and across the population as a whole. In figure 4, we present depictions of the contributions of the first three Cartesian PCA modes of the waveforms, with these modes corresponding to 76%, 7.5% and 6.6% of the total variance, respectively. The first PCA mode can be seen to generate the most significant component of the flagellar beat, an actuation perpendicular to the midline of the waveform. The second mode contributes compression and extension of the flagellum in the direction of the midline, also introducing a slight bend. Most recognizable in terms of its visual contribution to the waveform, the third mode introduces the characteristic sinusoidal shape to the beat, with the amplitude appearing to increase with the distance from the flagellum base.

Not only are the effects of the modes visually distinct, but their contributions differ over time in both magnitude and phase. Shown also in figure 4 are the average values of the first three PCA coefficients over a beating period, and we note that their magnitudes are comparable as the modes have been, without loss of generality, suitably normalized. The first mode, contributing 75% of the variance, is of much greater magnitude than the less significant components, thus it is the dominant contribution to the amplitude of the overall beat. Representing lesser contributions to both the variation and the beat amplitude, the coefficients of the higher-order modes are additionally out of phase, both with each other and the dominant mode. With the third mode generating the stereotypical sinusoid-like waveform, this phase difference implies that the characteristic shape of the beat lags behind the main oscillatory component, at least in this average waveform.

Repeating the above principal component analysis with waveforms represented as angle parametrizations yields analogous results, though with different principal components owing to the nonlinear transformation relating spatial data to angle form. It is not clear that these components can be separated into the constituents of the flagellar beat identified by analysis of the spatial data, highlighting that in general it may be of benefit to consider waveforms not only as reduced angle representations but also in spatial form, with the differing approaches potentially yielding different insights into the flagellar beat.

### 2.3. Assessing impacts of distal cytoplasmic blebbing

**Figure 6.**
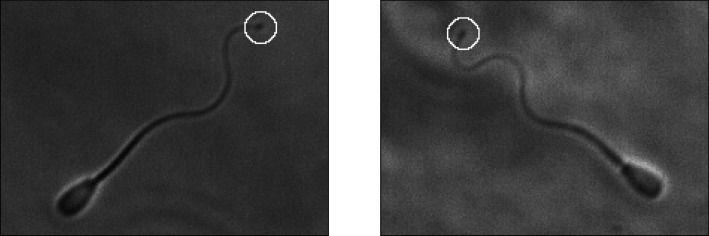
Visible signs of flagellar damage, with select frames taken from sample B. We see evidence of distal cytoplasmic blebbing, present here as darker, broader regions towards the tip of the flagellum, highlighted by an annotated white circle. In our sample of 137 swimmers that had undergone a freeze–thaw process, approximately one in four individual spermatozoa were found to exhibit visually identifiable blebbing.

With the spermatozoa in sample B having undergone a freezing and thawing process, we note that a significant proportion (25%) of these motile individuals exhibited visible signs of damage, specifically distal cytoplasmic blebbing. We show two example such swimmers in figure 6 and henceforth refer to swimmers with visible distal cytoplasmic blebbing as *blebbed*, with visually unaffected individuals being referred to as *unblebbed*. Having manually identified blebbed cells, we repeat the comparative analysis performed above for samples A and B on the motile blebbed and unblebbed subsamples.

We find that there is a statistically significant difference in the distributions of flagellum length between blebbed and unblebbed swimmers via a Kolmogorov–Smirnov test at the 0.5% significance level, although the same test is inconclusive for distributions of MDC and beating period. The Wilcoxon rank-sum test applied to the beating period does, however, suggest a difference in medians at the 5% significance level between blebbed and unblebbed periods, with the blebbed swimmers possessing a lower median compared to unblebbed cells. Finally, and consistent with inconclusive results pertaining to MDC, we find no statistically significant differences between the waveforms as a whole by considering linear model fits of the PCA coefficients. In turn, this statistically evidences that, despite the freeze–thaw transitions inducing distal flagellar blebbing in these bovine spermatozoa, the distal beating pattern of the blebbed spermatozoa is nonetheless preserved relative to those with no visible damage.

### 2.4. Waveform sampling

Using the rich dataset of captured spermatozoa beats, we propose a novel method for the generation of synthetic waveforms that follow the approximate distribution of digitized waveforms. Herein, *s* is an arclength parameter that has been rescaled for each individual swimmer by the flagellum length, so that *s* ranges from zero to one. Similarly, *t* denotes dimensionless time over a single beating period, with time having been normalized for each swimmer by their beating period so that here *t* also lies between zero and one (see Methods and material for full details). Working in the framework of angle parametrizations, fixing some integer *N*_*s*_, and uniformly sampling *N*_*s*_ waveforms $\theta_{1},\ldots ,\theta_{N_{s}}$ from our entire dataset, we can form a synthetic angle parametrization *S* via

2.2 $$S(s,t)=\left(1-\sqrt{N_{s}}\right)\mu (s,t)+\frac{1}{\sqrt{N_{s}}}\sum_{i=1}^{N_{s}}\theta_{i}(s,t)=\mu (s,t)+\frac{1}{\sqrt{N_{s}}}\sum_{i=1}^{N_{s}}[\theta_{i}(s,t)-\mu (s,t)],$$

parametrized by the normalized arclength *s* and rescaled time *t*, where *μ*(*s*, *t*) denotes the average of the angle parametrized waveforms in our dataset. Given a synthetic angle parametrization, we generate a waveform by uniformly sampling an arclength and period from the constructed dataset, together constituting a full description of a generated flagellar beat pattern. Synthetic waveforms created in this way retain the mean and variance of the original dataset, and hence approximate a representative spermatozoan waveform given approximate normality in the deviation of waveform angles from their mean. The latter assumption is informed by the distributions of PCA coefficients [32, ch. 2], shown in figure 2*c*,*d*. We include in the generated dataset, available as per the data access statement, an exemplar Matlab^®^ code for generating synthetic waveforms in this manner, with a sample synthetic beat being shown in figure 1*d*. Using the dataset of 216 spermatozoa presented here, this methodology is capable of producing over 10^60^ synthetic waveforms, with the number of possible outputs scaling exponentially with dataset size. It should be noted that application of this methodology to stratified datasets may necessitate sampling only from subsets of the data.

## 3. Discussion

In this work, we have produced and analysed a dataset of the flagellar beats of over 200 bovine spermatozoa and have described a methodology and pipeline that is easily integrated with phase contrast microscopy, which is ubiquitously available in theriogenological, andrological and cell motility laboratories. While also of pertinence to a broad biological community, we anticipate that the data generated in this study will also be of intense interest to the biophysical modelling community. Datasets generated with the same level of flagellar detail and scale have, to the best of our knowledge, been previously unavailable, with more of the distal flagellar tip being captured here than has been realized on large scales prior to this study, with the notable exception of the recent work of Daloglu *et al.* [21] that applied sophisticated holographic imaging to capture beats in three dimensions. Easily obtainable waveform data with the level of fidelity presented in this work has the potential to further the vast research area of heterogeneous population modelling, allowing the emergence of the next generation of evidence-based biophysical modelling for cell populations, spermatozoan behaviours in complex microenvironments, and more generally active matter physics. For example, the novel ability detailed here to generate synthetic waveforms will enable theoretical advances in understanding how realistic population heterogeneity impacts on the collective behaviours of active matter, while avoiding the need for the direct quantification of tens of thousands of flagellar beats for use in large-scale simulation studies.

The detailed digitization of flagellar beating and the widespread availability of such data additionally provides novel opportunities for quantitative comparison of flagellar beats across individuals, samples, and, in principle, species. Applying the comparative methodology described in this work to our two samples, we were able to identify statistically significant differences between the waveforms of the swimmers in each of the samples using linear model-fit parameters for the primary PCA coefficient, and thus examining more than simply coarse summary statistics such as period or wavelength, the latter of these not being uniquely defined in general. While the impacts of these quantitative differences on overall motility are not clear, differences in suggested surrogates of functional performance, such as MDC as a proxy for swimming efficiency, can be readily detected even in this initial study. Furthermore, future hydrodynamical and elastohydrodynamical computational studies could determine the extent of the effects, if any, of these dissimilar beats on swimmer progression and their metabolic demands, as well as their interactions with the microenvironment, including neighbouring swimmers. This example speaks more generally to the potential for quantitative and far-reaching comparisons of monoflagellated eukaryotic swimmers and their mechanical function with the availability of detailed, high-volume flagellar data, demonstrated here to be realizable.

Owing to the significant sample sizes that may be analysed via our presented methodology, intrasample variations and associated comparisons may also be explored, for instance cytoplasmic morphology and its correlates. In particular, cytoplasmic blebbing has been noted in freshwater fish sperm, for example sturgeon [33], and attributed to osmolar stress. Here we have also observed cytoplasmic blebbing of the distal tip for one in four sperm that has undergone cell freezing and thawing. Even with such a morphological dichotomy in the presence and absence of blebbing within the frozen sample (B), we were unable to differentiate between the spatial aspects of the waveforms exhibited by each subgroup of sample B, including the distal features, despite having amply large sample sizes to facilitate statistical power. This provides evidence that the damage inducing distal blebbing and the distal blebbing itself have no discernible effects on the beating characteristics of motile swimmers for reconstituted frozen bovine spermatozoa, with the exception of potential effects on beating period that warrant further exploration.

More generally, these example comparisons serve to illustrate how the presented methodology can be applied to assess the impacts, if any, of differences between swimmers on their overall motility function, using the details of highly resolved flagellar waveforms. We have demonstrated that such analysis, performed here automatically on sample sizes totalling hundreds of swimmers, and incorporating information from 90% of the flagellar length, is realizable with readily available imaging and computational techniques, and thus represents a viable framework for a new generation of detailed flagellar CASA, and more generally the advent of computer-assisted beat-pattern analysis (CABA). The introduction of traditional CASA has had a substantial impact in theriogenology, enabling systematic quality control and assurance in the livestock breeding industry [9], as well as fundamental cell science, with extensive fundamental studies of sperm function [11–14]. Further, the expansion into near-complete planar flagellar waveform capture offers the prospect of novel directions for the exploration of male gamete motility, and flagellated or ciliated organisms more generally. The significance of high-fidelity analysis of flagella is further highlighted by the recent work of Neal *et al.* [16], with the overall efficiency of a spermatozoon suggested to be linked to distal curvature. Measurement of such curvatures requires the capture of large proportions of the flagellum, greater than previously available en masse with readily accessible microscopy as demonstrated here to be achievable on significant sample sizes, though with further developments in imaging and subsequent image processing required to capture complete details of the distal flagellum. As an additional example, access to detailed flagellar data may also enable investigation into the symmetry of the spermatozoan beat, with significant asymmetry commonly associated with hyperactivation in mammalian spermatozoa but nevertheless exhibited at a much more subtle level here for bull in the absence of hyperactivation, as illustrated in figure 1*c*. Further, the method of quantitative analysis presented in this work need not be limited to the spermatozoa of any species, nor to single beating periods or to planar beats, with the detailed analysis of beating flagella relevant to a range of organisms, from the biflagellated alga *Chlamydomonas reinhardtii* to the helically driven bacterium *Escherichia coli*.

Datasets such as the one generated and presented in this work may additionally enable detailed investigation into the structure of the spermatozoan beat. In this study, we have seen that the population-average flagellar beat can be deconstructed into distinct components, with the primary contribution generating the large amplitude motion perpendicular to the midline, and the formation of the stereotypical sinusoid-like shape lagging behind. With detailed flagellar waveforms now available on a large scale, similar analyses may lead to a deeper understanding of the flagellar beat, suggested here for bovine spermatozoa but potentially applicable in more generality. If used in combination with mechanical measures and elastohydrodynamic models of the flagellum, for instance, extensive in-depth datasets of *in vitro* waveforms may facilitate explorations of the mechanical regulation and actuation of flagella, enabling data-driven queries of the validity of popular hypotheses of flagellar control [34–37].

In summary, we have investigated the beating characteristics of bovine spermatozoa, capturing more of the flagellar beat than previously acquired on such sample sizes without requiring sophisticated hardware or image analysis tools. With the resulting dataset publicly accessible, as described in the data access statement below, and with the prospect of readily producing further data, we have made available a wealth of kinematic data with potential for future use in in-depth quantitative studies that may lead to a deeper understanding of the flagellar beat, the biology of spermatozoa, and more generally active matter modelling. Indeed, such detailed waveform data will directly enable future biophysical modelling of heterogeneous swimmer populations, for example via the novel ability presented here to generate synthetic waveforms that retain key population characteristics. Most significantly, we have demonstrated that comparisons between the beating patterns of flagellated swimmers may be carried out quantitatively via non-standard application of dimensionality reduction methods, taking into account aspects of the flagellar beat that have previously been inaccessible or neglected. In doing so, we have showcased a realized pipeline for a new generation of computer-assisted flagellar analysis, readily achievable with current imaging and computational techniques. The presented approach leverages unprecedented flagellar fidelity with non-specialized imaging modalities to assess and classify the intricacies of the spermatozoan beat, facilitating potential future study into individual motility, viability, function and variability in motile flagellate populations.

## 4. Methods and material

Details of sample preparation, image capture, waveform tracking and smoothing can be found in the accompanying electronic supplementary material.

### 4.1. Analysis and parametrization of flagella

#### 4.1.1. Determining the beating period

While existing CASA implementations are able to approximate the beating period of spermatozoa from the oscillations of the body over time, as first suggested by Schoëvaërt-Brossault [38] and Serres *et al.* [39], we opt to determine the period of the flagellar beat using the captured flagellar kinematics. The recent work of Gallagher *et al.* [15] also based their estimate of the period on captured flagellar data, though implicitly required that there were only two local extrema of curvature at a given material point over a beating period. Here we avoid this requirement by computing the period using the autocorrelation of a material point, which may be thought of as tracking the location of this point over time and identifying the time after which its trajectory is most self-similar. Performing this computation using multiple points along the flagellum, the minimal beating period of each flagellar waveform may be automatically identified, which we denote by *T*, with results verified by eye. We note that sampling each flagellum only at its midpoint was sufficient in this case to reliably determine the beating period.

#### 4.1.2. Normalization and discretization

Here, the results of spatial and temporal smoothing are natural Cartesian representations of each flagellar waveform, denoted $\left(\tilde{x}(\tilde{s},\tilde{t}),\tilde{y}(\tilde{s},\tilde{t})\right)$, where for each individual the arclength, $\tilde{s}$, ranges from zero to the captured flagellum length, *L*, and $\tilde{x}$, $\tilde{y}$ are relative to the sperm reference frame, as detailed in the electronic supplementary material and as used in figure 1*c*. The time, $\tilde{t}$, encompasses the timestamps of the captured frames containing this flagellum, with the first frame occurring at $\tilde{t}=0$ without loss of generality, and the timestamp of the final frame denoted *T*_*f*_. Here, we normalize each waveform in space by the length of the flagellum, so that the rescaled arclength, $s=\tilde{s}/L$, lies between zero and one for all of the swimmers. While this rescaling of arclength is here justified by the tight clustering of measured flagellum length about its mean, as shown in figure 5, we note that in the absence of such a justification one may still consider waveforms normalized in this way, with subsequent comparisons between individual swimmers being made in terms of rescaled arclength *s*. Alternatively, the approaches presented in this work may be readily adapted to consider waveforms that have not been normalized in space, for example with beat patterns being stratified by flagellum length or spatially truncated where appropriate, enabling like-for-like comparisons of material points along the flagellum even when flagellum length may vary significantly amongst the population. Here, the spatially normalized waveforms are then discretized at 1000 points in space, capturing at each instant the locations of material points equally spaced along the flagellum at fixed arclengths *s*_*i*_ = (*i* − 1)/999, *i* = 1, …, 1000.

Similarly, the temporal dynamics of each swimmer are rescaled by their beating period, with rescaled time being denoted $t=\tilde{t}/T\in [0,T_{f}/T]$ and the fully normalized waveform written as (*x*(*s*, *t*), *y*(*s*, *t*)). Before being discretized in time, each waveform is temporally truncated such that it represents only a single beat of the captured flagellum, i.e. it is restricted to some interval $t\in [t^{⋆},t^{⋆}+1)$, noting that the period of the rescaled beat is one. The offset *t*^⋆^ is found for each swimmer by comparing the locations of the flagellum midpoint (*x*(*s*_500_, *t*), *y*(*s*_500_, *t*)) at times *t* and *t* + 1 for all valid *t* ∈ [0, *T*_*f*_/*T*], selecting *t*^⋆^ so as to minimize the Euclidean distance between the locations of the material point at each instant. The temporally truncated waveforms are then discretized into 100 equispaced timepoints, and all waveforms are aligned in phase with one another by prescribing the location of a fixed phase in the periodic behaviour, with this simple method of phase alignment being verified *a posteriori*. While here we have truncated captured data to a single beating period, the methodology presented in this work may be readily extended to consider multiple flagellar beats, enabling quantification of intra-individual beat pattern variability.

#### 4.1.3. Representations of the flagellum

Above we have described normalized waveforms using a natural representation of the rescaled flagellar beat in spatial Cartesian coordinates. However, in some cases it is more convenient to consider each beat in terms of its tangent angle parametrization, for example when generating synthetic beating patterns. This tangent angle parametrization, here denoted by *θ*(*s*, *t*), is defined by the nonlinear relation

4.1 $$\theta (s,t):=\arctan\left(\frac{y_{s}(s,t)}{x_{s}(s,t)}\right),$$

where here *y*_*s*_(*s*, *t*) and *x*_*s*_(*s*, *t*) denote the derivatives of *y*(*s*, *t*) and *x*(*s*, *t*) with respect to normalized arclength *s*, and arctan is the four-quadrant inverse tangent function (e.g. the function atan2 in Matlab^®^). This relation is readily inverted to recover the Cartesian beating pattern from the tangent angle in the reference frame of the spermatozoon, as described in the section on flagellum smoothing in the electronic supplementary materials and illustrated in figure 1*c*

4.2 $$(x(s,t),y(s,t))=\int_{0}^{s}(\cos\theta (\hat{s},t),\sin\theta (\hat{s},t))\;\text{d}\hat{s}.$$

In practice, these derivatives, integrals, and function evaluations must be performed numerically, and we observe negligible cumulative error when transforming between parametrizations using 1000 material points to represent each flagellum.

### 4.2. Waveform analysis

#### 4.2.1. Principal component analysis

A standard tool for dimensionality reduction in various contexts, PCA may be used to identify the most significant constituents of the flagellar beat. Given multiple observations of a quantity, which in our case will either be *xy* coordinates or the accompanying tangent angle parametrization of material points along the flagellum, PCA outputs time-independent components or *modes*, along with time-dependent *coefficients*, with the coefficients for the *i*th mode here being denoted *c*_*i*_. These PCA modes and coefficients are given in descending order of their contributions to the variance of the observed quantities, with their contributions quantified by *weights* that sum to unity. For a technical description of PCA, we direct the interested reader to the relevant work of Werner *et al.* [25]. In the study presented here, at a timepoint *t*_*j*_ our observations will be of the form (*x*_1_, *y*_1_, …, *x*_1000_, *y*_1000_) or (*θ*_1_, …, *θ*_1000_), corresponding to Cartesian and tangent angle representations of the waveform, respectively, where *x*_*i*_ denotes *x*(*s*_*i*_, *t*_*j*_) for the *i*th equispaced arclength *s*_*i*_ at the *j*th timepoint *t*_*j*_, with analogous definitions for *y*_*i*_ and *θ*_*i*_.

PCA in the context of flagellar analysis is often performed on a set of observations taken from an individual cell, yielding for that individual a set of modes, coefficients and weights. While this may provide insight into the constituent components of a particular waveform, its utility in comparing the beats of different swimmers is limited to consideration of the PCA modes, with the coefficients not being comparable due to their ties to their respective, and different, modes. Further, as modes corresponding to individual swimmers can represent different contributions to the overall beating, given by their individual weights, comparisons between the modes of different swimmers are themselves very limited in scope. Thus, in this work we apply principal component analysis to all the swimmers in a given sample at once, combining the observations of the motion of each material point across the population. This enables us to comment not only on individual variation, but on the quantitative variation of waveforms across the population, with the PCA coefficients now comparable between swimmers as they are each with respect to a single common set of modes. Modes are normalized so as to render comparison between the coefficients of different modes meaningful. For the dataset generated in this work, the three most prominent Cartesian PCA modes for the population as a whole cumulatively capture over 90% of the overall variance over the population, while the median proportion of the variance of individual waveforms captured by these three modes is 82%.

#### 4.2.2. Maximal distal curvature

Given a tangent angle parametrization *θ*(*s*, *t*) of a flagellar waveform, the signed curvature, *κ*, is given by *κ* :*= θ*_*s*_(*s*, *t*), where again the subscript denotes a derivative with respect to normalized arclength. Numerically computing this curvature over the distal 10% of the captured flagellum, we take the maximum of its absolute value over this distal flagellar portion and the entire beating period, and report this quantity as the MDC. With the data in this study capturing all but the most distal 10% of the observed flagellum, with processed data screened by this requirement, this measure is therefore computed over 81% and 90% of the true flagellum length and thus may not represent the true curvature of the most distal tip of the beating flagellum. However, the MDC as defined here captures details of the distal flagellar region previously unavailable on this scale, with the distal portion of the flagellum recently suggested to be correlated with overall swimming efficiency [16].

### 4.3. Statistical tests

Distributions of the first Cartesian PCA coefficient are compared via linear least squares fitting of the coefficient for each individual swimmer, where the linear model to be fitted is

4.3 $$c_{1}=\alpha +\beta \cos\left(kt-\phi \right),$$

informed by the approximately sinusoidal form of the average coefficient shown in figure 2. Here, *α* and *β* are constant in arclength and time, but vary between sperm. In addition, *t* again represents normalized time, and wavenumber and phase parameters *k* and *ϕ* are first approximated by nonlinear least-squares fitting of the mean coefficient, ${\bar{c}}_{1}$ (figure 4*b*,*d*,*f*). This is implemented in Matlab^®^, in particular keeping both *k* and *ϕ* fixed throughout the linear fits. Differences between fits are quantified by standard two-tailed two-sample Kolmogorov–Smirnov (KS) tests performed on the distributions of the parameters *α* and *β*, with the conclusion of an overall significant difference drawn only if both tests are significant at the 5% level. Refinements are possible on larger sample sizes using the joint distribution of fit parameters, though are not implemented in this work. All other statistical tests performed are either KS tests or Wilcoxon rank-sum tests, each at the 5% significance level.

## Supplementary Material

Supplementary Materials

Reviewer comments

## Data accessibility

The data used and generated in this paper is freely available from the University of Oxford Research Archive (ORA) via the link http://dx.doi.org/10.5287/bodleian:dpw9XANJa.
