# Supplementary material for: Computer-assisted beat-pattern analysis and the flagellar waveforms of bovine spermatozoa
Source: R Soc Open Sci. 2020 Jun 17;7(6):200769. doi: 10.1098/rsos.200769 (PMC7353979; doi:10.1098/rsos.200769)
Supplement: Supplementary Materials [file rsos200769supp1.pdf]

Computer-assisted beat-pattern analysis and the flagellar waveforms of bovine spermatozoa – Supplementary Material

Benjamin J. Walker*, Shiva Phuyal, Kenta Ishimoto, Chih-Kuan Tung, Eamonn A. Gaffney
* benjamin.walker@maths.ox.ac.uk

1 Sample preparation & image capture

1.1 Reagents & media

Tyrode Albumin Lactate Pyruvate (TALP) medium [1] was used as a standard medium for bovine sperm. TALP comprised of 3.1 mM KCl, 25 mM $NaHCO_3$, 10 mM HEPES Free Acid, 99 mM NaCl, 0.39 mM of NaH_2PO_4, 25.4 mM of Na-lactate, 2 mM of $CaCl_2$, 1.1 mM of $MgCl_2$, 0.11 mg/mL Sodium pyruvate, 5μg/mL of gentamycin and 6 mg/mL of Bovine Serum Albumin. The final pH value of the TALP solution was 7.4. 1% (w/v) of Methyl Cellulose (MC, 4000 cP at 2%) solution was made by slowly stirring MC powder in TALP in room and chilled temperatures alternately. All fluids were equilibrated in a 38.5°C incubator (bovine body temperature) with 5% CO_2 in humidified air before use. Rheology of the MC solutions were measured using a rotational shear rheometer (TA Instruments, DHR3) with a standard cup and DIN rotor at 38.5°C in oscillation mode. The measured moduli are shown in fig. S1. At the frequency of 10 Hz, the storage modulus, G', and loss modulus, G'', were measured as $G' = 0.73$ Pa and $G'' = 5.37$ Pa, respectively. As far as considering the dynamical frequency being less than 20 Hz, the rheological data were well approximated by the viscoelastic Maxwell model, from which we estimated the effective viscosity, 0.088 Pa·s, and the relaxation time, 0.0014 s.

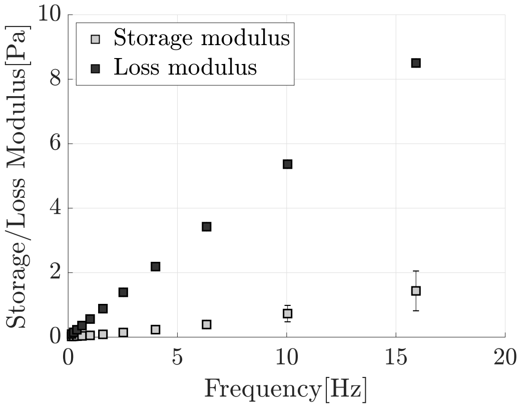

Figure S1: Measurements of storage and loss moduli for the methyl cellulose medium at various frequencies. We observe that both the storage and loss moduli, shown as light and dark squares respectively, are well-approximated by a linear viscoelastic Maxwell model. Error bars corresponding to the standard deviation of measurements are shown for each of the moduli, though only two such bars are visible at the resolution of this figure.

1.2 Sample preparation

All bovine semen samples were kindly provided by Genex Cooperative, Inc. Sample A was a fresh semen sample from one bull, from which the beats of 79 sperm were captured. Shortly after semen collection, 1 mL of bovine semen sample was diluted in 5 mL of warm TALP, and then processed identically to Tung et al. [2]. Sample B was a frozen semen sample from another bull, from which the beats of 137 sperm were captured. Notably, as Samples A and B differ in both their handling and their source animal, we will not seek to draw biological conclusions from any further observed distinctions between them, and place emphasis wholly on the comparative methodology presented in this work that is exemplified by the consideration of these two samples. After collection, the semen was first diluted in OptiXcell extender (IMV t Technologies, L'Aigle, France), and then frozen according to the standard procedures followed at Genex [3]. The thawing process is similar to our previous procedures for thawing frozen semen straws [4]. The frozen semen was first thawed in a water bath at 37°C for 30 seconds, and then transferred on top of two layers (40% and 80%) of BoviPure diluted in BoviDilute. The live sperm were separated from the rest of the fluid by centrifugation (100 x g for 10 minutes), as they formed a pallet at the bottom of the tube. After removal of the supernatant, 3 mL of TALP was added and followed by another centrifugation (100 x g for 3 minutes) to wash the sperm pallet. 100 μL of TALP was

added to the sperm pallet to make the suspension, which was kept in an incubator maintained at 38.5°C with 5% of CO_2.

1.3 Image acquisition and experimental setup

Our previously developed microfluidic device [5] was adapted for imaging to create a well-controlled, no-flow environment. Before the experiments, devices were filled with a 1% MC solution and kept for at least 2 hours in an incubator at 38.5°C with 5% of CO_2. Sperm suspension was then seeded onto the 2 mm access hole of the device and sperm were allowed to swim inside the device. The microfluidic chamber is 100 μm deep, and we captured videos close to the lower surface by a scientific CMOS camera (ANDOR Neo for sample A (fresh) and Zyla for sample B (frozen)) at their respective highest frame rates (135-165 frames per second), in conjunction with an inverted microscope with phase contrast and NIS-Elements software. Given an estimated 20 μm depth of the focal plane, only vague shadows of sperm swimming close to the upper surface were seen. The devices were maintained at 38.5°C during the experiments.

2 Tracking and smoothing of flagellar waveforms

2.1 Tracking and selection

Videomicroscopy data is processed using the TrackMate plugin included in the popular software package Fiji [6–8], with TrackMate automatically tracking the locations of the spermatozoa between frames by identifying their cell bodies. Individual swimmers are then isolated using bespoke Fiji macros, utilising automatic local thresholding to segment individual swimmers into binary masks. Typically, swimmers were approximately one cell length away from other individuals. In detail, written in the form of the ImageJ Macro language, the preprocessing performed constituted of the following operations:

```
run("32-bit");
im = getImageID();
run("Z Project...", "projection=[Average Intensity]");
avg = getImageID();
imageCalculator("Divide stack 32-bit",im,avg);
selectImage(avg);
run("Close");
selectImage(im);
run("Smooth", "stack");
run("Unsharp Mask...", "radius=5 mask=0.80 stack");
run("Enhance Contrast...", "saturated=0.3 normalize process_all");
run("Remove Outliers...", "radius=5 threshold=50 which=Bright stack");
run("Minimum...", "radius=1 stack");
run("8-bit");
run("Auto Local Threshold", "method=Phansalkar radius=15 parameter_1=0 parameter_2=0 stack");
```

These processing steps correspond to a background subtraction, smoothing and contrast-enhancing, noise reduction, and local thresholding to produce a binary mask. We note that bespoke preprocessing of swimmers into a binary mask is likely required for other datasets. Each swimmer mask is then processed using the fully automated scheme of Walker et al. [9], with flagella being identified by their approximately consistent visible width. The resulting digital representations of the flagella, validated by eye and consisting of pixel locations of the flagellum in each frame, are screened to accept only those where the observable flagellum length, projected onto the focal plane of the microscope, varied by less than 10% of the maximum over multiple beating periods. Of these accepted individuals, frames containing digitised flagella of captured length greater than this 90% threshold were truncated in space to enforce uniformity whilst avoiding the need for extrapolation. This truncated flagellar length represents the true flagellar length to within approximately 10%. Reliably reporting the true flagellar length is precluded by difficulties in imaging the most-distal region of the flagellum, an issue common in the microscopy of flagellates though here limited to approximately the distal 10%.

2.2 Spatial and temporal smoothing

With the data by construction reporting spatiotemporal information for approximately 90% of the visible flagellum for each swimmer, pixel locations are identified with Cartesian xy coordinates, translated and rotated so that the proximal flagellar end is both at the origin and aligned along the horizontal axis $(1, 0)$, as illustrated in Figure 1 of the main text (lower left), hence quantifying flagellar motion relative to the swimmer body. Each flagellum is parameterised by arclength and time, and smoothing in both space and time performed using smoothing splines and Gaussian convolutions, respectively, in the software package MATLAB®. Owing to infrequent erroneous results of the flagellar identification and tracking process, a low proportion (less than 10%) of frames corresponding to each individual swimmer are omitted from temporal smoothing due to such errors.

Instead, for these rare frames, flagellum data is linearly interpolated from the preceding and following frames, and we note in particular that the frame rates are sufficiently high so as to preclude significant interpolation errors. All smoothing and interpolation is validated by visual comparison with the unsmoothed data.

In more detail, the raw Cartesian coordinates of captured waveforms were first smoothed in space using the MATLAB `smoothingspline` fit type with smoothing parameter 0.005. Waveforms were then resampled at 1000 material points up to 90% of the maximum recorded flagellum length. The effects of noise on this computed maximum length were first reduced by taking a moving arithmetic mean with a window size of 60 frames. Beating patterns were then aligned such that in a single frame the base of the flagellum was parallel to some fixed axis, here the Cartesian x-axis. Subsequent frames were then reoriented so as to minimise the sum of the squared differences of the coordinates of the proximal 20% of the flagellum, verified by eye in all cases to successfully align the proximal regions of the flagellum.

Temporal smoothing then proceeded by the interpolation of any missing frames due to noted infrequent errors in the tracking and spatial alignment process via linear interpolation using `scatteredInterpolant`. This interpolation was performed on the smoothed angle parameterisation of the waveform, itself parameterised by 1000 material points and typically hundreds of frames, with smoothing taking place via a MATLAB default Gaussian filter with normalised standard deviation of unity. Following interpolation, a circular disk averaging filter of dimensionless radius two was convolved with the angle parameterisation, itself parameterised by discrete frames and arclengths. The approximate beating period and frames over which such periodic beating occurs were calculated from this smoothed data using the script `find_period.m`, as may be accessed according to the data access statement found in the main text, following the approach as described in the main text. The angle parameterisation was then truncated in time over this period and resampled at 100 timepoints via cubic interpolation. Finally, a smoothing Gaussian filter of normalised standard deviation five was convolved with the approximately periodic resampled angle parameterisation, padding circularly in time and replicating in space. The results of spatial and temporal smoothing, including period identification, were verified by eye in all cases.

We note that the methodologies presented in the main text for comparative quantitative waveform analysis and synthetic waveform generation are not reliant on the details of the smoothing used in this study, and may be generically applied to captured waveform data.

References

[1] Parrish JJ, Susko-Parrish J, Winer MA, First NL. Capacitation of bovine sperm by heparin. Biology of Reproduction. 1988;38:1171–1180.

[2] Tung Ck, Lin C, Harvey B, Fiore AG, Ardon F, Wu M, et al. Fluid viscoelasticity promotes collective swimming of sperm. Scientific Reports. 2017;7(1):3152.

[3] Kaproth MT, Rycroft HE, Gilbert GR, Abdel-Azim G, Putnam BF, Schnell SA, et al. Effect of semen thaw method on conception rate in four large commercial dairy heifer herds. Theriogenology. 2005;63(9):2535 – 2549.

[4] Tung Ck, Ardon F, Fiore AG, Suarez SS, Wu M. Cooperative roles of biological flow and surface topography in guiding sperm migration revealed by a microfluidic model. Lab Chip. 2014;14(7):1348–1356.

[5] Tung Ck, Ardon F, Roy A, Koch DL, Suarez SS, Wu M. Emergence of Upstream Swimming via a Hydrodynamic Transition. Physical Review Letters. 2015;114(10):108102.

[6] Schneider CA, Rasband WS, Eliceiri KW. NIH Image to ImageJ: 25 years of image analysis. Nature Methods. 2012;9(7):671–675.

[7] Schindelin J, Arganda-Carreras I, Frise E, Kaynig V, Longair M, Pietzsch T, et al. Fiji: an open-source platform for biological-image analysis. Nature Methods. 2012;9(7):676–682.

[8] Tinevez JY, Perry N, Schindelin J, Hoopes GM, Reynolds GD, Laplantine E, et al. TrackMate: An open and extensible platform for single-particle tracking. Methods. 2017;115:80–90.

[9] Walker BJ, Ishimoto K, Wheeler RJ. Automated identification of flagella from videomicroscopy via the medial axis transform. Scientific Reports. 2019;9(1):5015.
